# The chemokine receptor CX_3_ CR1 coordinates monocyte recruitment and endothelial regeneration after arterial injury

**DOI:** 10.15252/emmm.201707502

**Published:** 2017-12-11

**Authors:** Tobias Getzin, Kashyap Krishnasamy, Jaba Gamrekelashvili, Tamar Kapanadze, Anne Limbourg, Christine Häger, L Christian Napp, Johann Bauersachs, Hermann Haller, Florian P Limbourg

**Affiliations:** ^1^ Vascular Medicine Research Hannover Medical School Hannover Germany; ^2^ Department of Nephrology and Hypertension Hannover Medical School Hannover Germany; ^3^ Department of Cardiology and Angiology Hannover Medical School Hannover Germany; ^4^ Present address: Institute of Radiology Hannover Medical School Hannover Germany; ^5^ Present address: Department of Plastic, Aesthetic, Hand and Reconstructive Surgery Hannover Medical School Hannover Germany; ^6^ Present address: Institute for Laboratory Animal Science and Central Animal Facility Hannover Medical School Hannover Germany

**Keywords:** CX_3_CR1, endothelial cells, monocytes, regeneration, vascular injury, Cardiovascular System, Immunology

## Abstract

Regeneration of arterial endothelium after injury is critical for the maintenance of normal blood flow, cell trafficking, and vascular function. Using mouse models of carotid injury, we show that the transition from a static to a dynamic phase of endothelial regeneration is marked by a strong increase in endothelial proliferation, which is accompanied by induction of the chemokine CX_3_ CL1 in endothelial cells near the wound edge, leading to progressive recruitment of Ly6C^lo^ monocytes expressing high levels of the cognate CX_3_ CR1 chemokine receptor. In *Cx3cr1*‐deficient mice recruitment of Ly6C^lo^ monocytes, endothelial proliferation and regeneration of the endothelial monolayer after carotid injury are impaired, which is rescued by acute transfer of normal Ly6C^lo^ monocytes. Furthermore, human non‐classical monocytes induce proliferation of endothelial cells in co‐culture experiments in a VEGFA‐dependent manner, and monocyte transfer following carotid injury promotes endothelial wound closure in a hybrid mouse model *in vivo*. Thus, CX_3_ CR1 coordinates recruitment of specific monocyte subsets to sites of endothelial regeneration, which promote endothelial proliferation and arterial regeneration.

## Introduction

Maintenance and restoration of endothelial integrity are critical for blood vessel function. Endothelial cells (EC) form a monolayer in the inner surfaces of blood vessels that controls exchange of metabolites and regulates coagulation and cell trafficking. Cardiovascular diseases, such as atherosclerosis, vascular interventions, or bypass surgery, cause EC damage or overt defects in the endothelial monolayer, which triggers vascular inflammation, neointima formation, and ultimately vessel obstruction if endothelial integrity is not restored (Gimbrone & Garcia‐Cardena, 2016).

Under physiological conditions, EC replication is inhibited by cell contact and laminar flow (Akimoto *et al*, 2000; Chen *et al*, 2000). The loss of few cells is repaired rapidly by extension and spreading of adjacent EC without the need for proliferation (Reidy & Schwartz, 1981). However, larger EC lesions require proliferation to regenerate the endothelial monolayer and prevent neointima formation (Haudenschild & Schwartz, 1979). *In vivo,* proliferation is initiated near the wound edge but subsequently spreads to areas distant from the wound (Filipe *et al*, 2008), which contrasts with *in vitro* EC wound models, in which the zone of proliferation is narrow and restricted to the immediate wound edge (Chen *et al*, 2000), suggesting the contribution of so far unrecognized cellular interactions and mechanisms in the coordination of EC proliferation *in vivo*.

Vascular injury leads to a regulated inflammatory response, during which neutrophils and monocytes are recruited by chemokines (Koenen & Weber, 2011). The CX_3_CL1‐CX_3_CR1 axis is a critical regulator of the vascular injury response (Schafer *et al*, 2004; Flierl *et al*, 2015). CX_3_CL1 (fractalkine) is a membrane‐bound chemokine expressed by activated EC after injury (Bazan *et al*, 1997; Liu *et al*, 2006), which binds to its cognate receptor CX_3_CR1 expressed on monocytes, subsets of NK cells and T cells (Jung *et al*, 2000), and which mediates preferential arrest of monocytes and adhesion to EC under flow (Fong *et al*, 1998; Ancuta *et al*, 2003).

A minor subset of monocytes with patrolling behavior, called Ly6C^lo^ monocytes in mice and which corresponds to the non‐classical subset of human monocytes, shows high levels of CX_3_CR1 and interacts constitutively with blood vessels, where under high flow conditions, this subset is preferentially recruited to activated EC by CX_3_CL1 (Ancuta *et al*, 2003; Schulz *et al*, 2007). In the steady state, Ly6C^lo^ monocytes originate from inflammatory Ly6C^hi^ monocytes in a process called “monocyte conversion” (Yona *et al*, 2013; Gamrekelashvili *et al*, 2016). They are long‐lived and crawl along the luminal side of EC to monitor blood vessels and scavenge microparticles (Auffray *et al*, 2007), a feature shared with the human non‐classical monocyte subset (Cros *et al*, 2010). In kidney models of endothelial injury, Ly6C^lo^ monocytes orchestrate EC clearance through interaction with subsets of activated EC in a CX_3_CL1‐ and CX_3_CR1‐dependent manner (Carlin *et al*, 2013). Ly6C^lo^ monocytes were also suggested to contribute to ischemic tissue repair (Nahrendorf *et al*, 2007). Of note, monocytes have recently been reported to induce EC proliferation *in vitro* (Schubert *et al*, 2008) and to participate in vascular repair after vascular injury *in vivo* (Becher *et al*, 2014). Furthermore, Ly6C^lo^ monocytes confer endothelial protection in atherosclerosis models (Quintar *et al*, 2017).

We studied the relevance of CX_3_CR1 for monocyte subset recruitment and endothelial regeneration in a model of perivascular carotid electric injury (CI), which is particularly tailored to study aspects of endothelial re‐endothelialization (Carmeliet *et al*, 1997).

## Results

### Endothelial proliferation coincides with CX_3_CL1 induction

**Figure 1:**
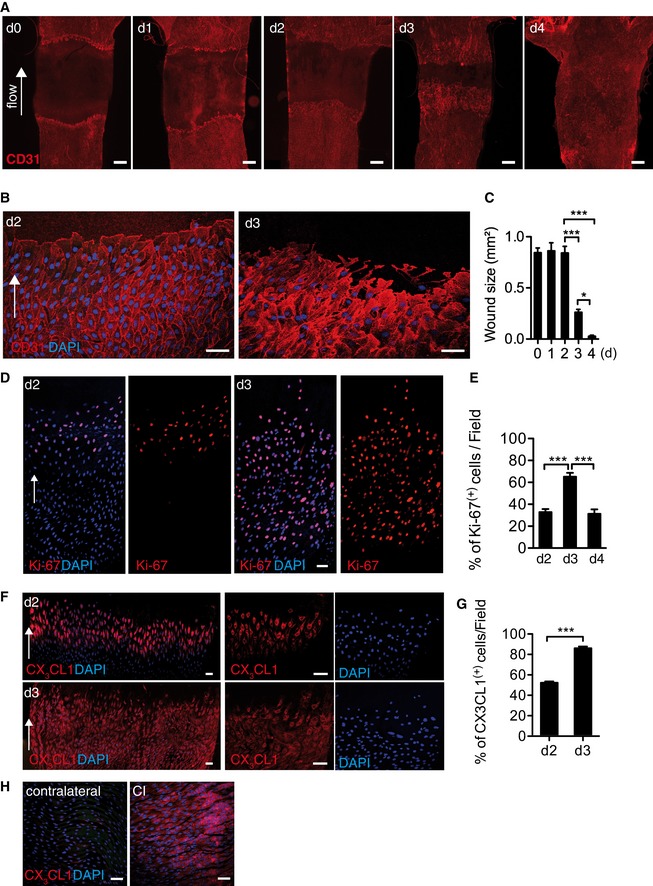
Endothelial behavior *in vivo* after carotid injury En face fluorescence microscopy of immunostained carotid arteries after CI. Scale bar: 400 μm.Composite confocal images of endothelial wound edge. Scale bar: 75 μm.Kinetics of wound closure post‐CI (d0 *n* = 9, d1 *n* = 8, d2 *n* = 6, d3 *n* = 5, d4 *n* = 15).Confocal images of immunostained carotids post‐CI. Scale bar: 75 μm.Kinetics of endothelial cell proliferation (d2 *n* = 24, d3 *n* = 12).Composite confocal immunostained images after injury (left), scale bar: 150 μm. Single field images of individual channels (right), scale bar: 75 μm.Quantitative analysis of CX_3_CL1 expression in the injury area. *n* = 5 carotids/group.Confocal images of healthy or injured endothelium, scale bar: 75 μm.Data information: White arrows indicate the direction of flow of blood. Data are presented as mean ± SEM. Statistical analysis: (C, E) one‐way ANOVA with Bonferroni's multiple‐comparison test (***P < 0.001, *P < 0.05); (G) two‐tailed Student's unpaired *t*‐test, *P* < 0.0001.

We first analyzed the endothelial monolayer in whole‐mount carotids en face with confocal microscopy at different time points after CI. During the first 2 days after CI, the endothelial wound remained static and EC in the wound border appeared regularly shaped and densely aligned in an orderly fashion (Fig 1A–C). After day 2, a switch to a dynamic phase of endothelial wound closure occurred, which resulted in restoration of endothelial integrity at day 4 (Fig 1C). During the dynamic phase, EC appeared enlarged and irregular with filopodia extending into the wound (Fig 1A and B).

**Figure EV1:**
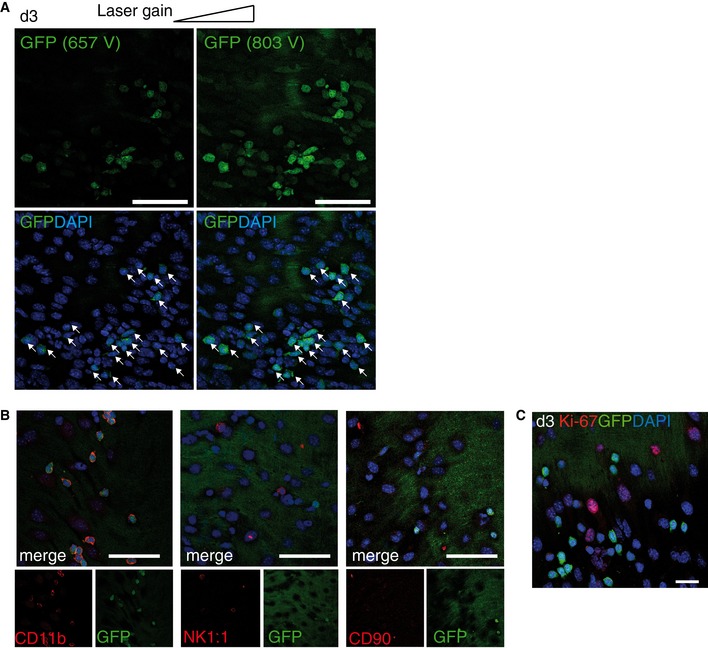
Identification and characterization of GFP ^+^ cells by confocal microscopy Confocal images showing infiltration of wound area by Ly6C^lo^GFP^hi^ cells (d2, static phase; d3, dynamic phase). Employing the technique of using high/low laser gain, we observed that the majority of the cells in the injured area are Ly6C^lo^GFP^hi^ cells (white arrows) that fluoresce under low laser gain (657 V). Scale bars: 30 μm.Confocal images of carotid arteries 3 days postinjury stained for NK1.1 and CD90.2. The images in the lower inlay contain red (NK1.1/CD90) and green (GFP) channels. Scale bar: 75 μm.Confocal image of proliferation depicts Ki‐67 expression in the proximal wound edge is specific to GFP^−^ cells (merge) (red, Ki‐67; green, GFP; blue, DAPI). Scale bar: 150 μm.

Endothelial cells proliferation is not observed during the first 36 h after injury but is noted after 50 h (Filipe *et al*, 2008). By Ki‐67 staining, EC proliferation at day 2 was low and restricted to the immediate wound edge, but increased dramatically at day 3, due to an expansion of the proliferation area, and ceased with wound closure at day 4 (Fig 1D and E). Furthermore, proliferation was restricted to resident EC (Fig EV1C). This confirms earlier reports of EC proliferation during wound closure (Schwartz *et al*, 1978; Filipe *et al*, 2008), but also defines the temporal pattern of two distinct phases of endothelial regeneration, characterized by progressive endothelial proliferation.

Interestingly, during the static phase of EC regeneration, CX_3_CL1 expression was induced but restricted to EC near the injury site (Fig 1F and H). However, CX_3_CL1 expression area expanded markedly during the dynamic phase of endothelial regeneration, involving EC further away from the wound edge (Fig 1F and G), which recapitulated the spatiotemporal distribution of endothelial proliferation during EC regeneration.

### Recruitment of Ly6C^lo^ monocytes during endothelial regeneration

**Figure 2:**
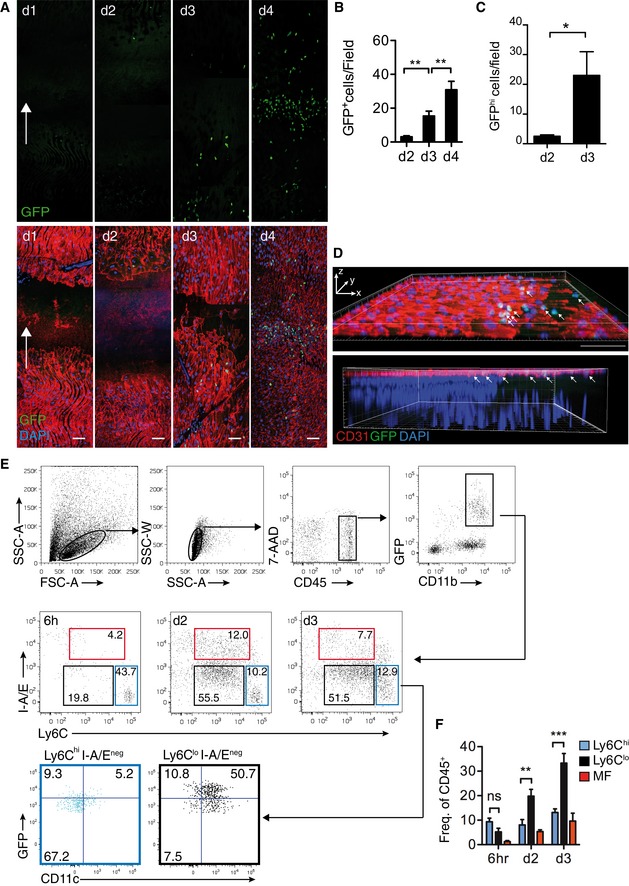
Ly6C^lo^ monocyte recruitment after arterial injury Composite confocal images of carotid arteries from *Cx3cr1* ^*GFP/+*^ mice after injury. Top panel displays GFP channel. Scale bar: 75 μm.Quantification of GFP^+^ cells/field in the proximal wound (d2 *n* = 15, d3 *n* = 12, d4 *n* = 10).Quantification of GFP^hi^ cells/field after injury (d2 *n* = 4, d3 *n* = 5).3D reconstruction of serial confocal images postinjury from *Cx3cr1* ^*GFP/+*^ mice (d3). Scale bar: 75 μm. Arrows indicate dimensional axis.Representative flow cytometry of single cell suspension from injured carotids (6 h *n* = 3, d3 *n* = 5, d3 *n* = 3).Quantitative analysis of cell populations, depicted as frequency of CD45^+^PI^−^ (live, 6 h *n* = 3, d3 *n* = 5, d3 *n* = 3).Data information: White arrows indicate the direction of flow of blood. Data are presented as mean ± SEM. Statistical analysis: (B, F) one‐way ANOVA with Bonferroni's multiple‐comparison test (***P < 0.001, **P < 0.01); (C) two‐tailed Student's unpaired *t*‐test, *P = 0.0314.

**Figure 3:**
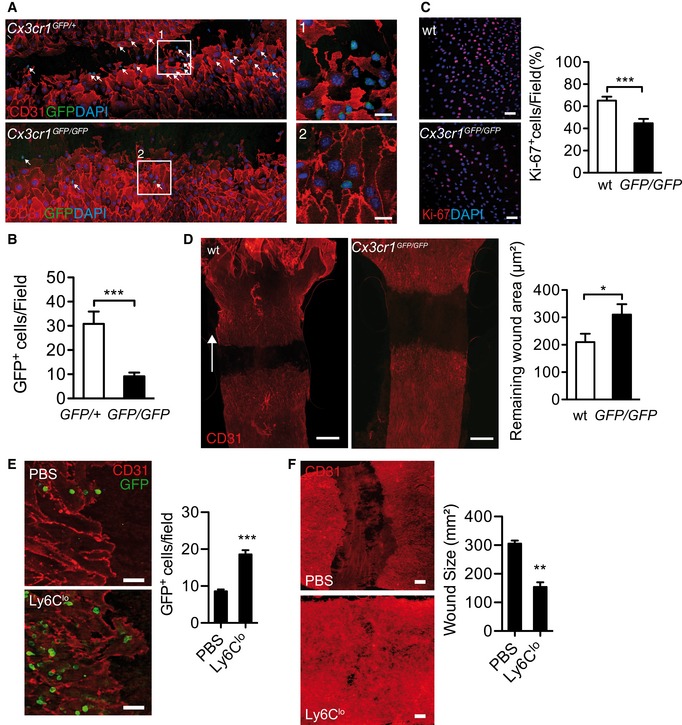
*Cx3cr1* loss of function impairs EC regeneration Composite confocal images of immunostained carotid arteries after injury (left), single close‐up of insert (right). Scale bar: 75 μm. Arrows point to GFP^+^ cells.Quantification of GFP^+^ cells/field in the proximal wound area (*n* = 10/12).Representative immunostained images of proximal wound (left), scale bar: 75 μm. Quantification of EC proliferation (Ki‐67^+^ cells/DAPI^+^ cells/field, right). *n* = 12/18.EC wound healing in WT and *Cx3cr1* ^*GFP/GFP*^ mice (d3). Scale bar: 400 μm. White arrow indicates the direction of flow of blood. Representative immunostained image (left) and quantification of remaining wound area (between two wound fronts, right). WT *n* = 16, *Cx3cr1* ^*GFP/GFP*^ *n* = 9.Representative confocal images of wound edge (left) and quantification of GFP^+^ cells (right) after injection of PBS or 1 × 10_6_ Ly6C^lo^ monocytes from *Cx3cr1* ^*GFP/+*^ donors into *Cx3cr1* ^*GFP/GFP*^ recipients after CI. *n* = 4/4. Scale bar: 200 μm.Representative immunostained images of wound area (left) and quantification of wound size (right) after injection of PBS or Ly6C^lo^ monocytes. *n* = 4/4. Scale bar: 200 μm.Data information: Data are presented as mean ± SEM. Statistical analysis: (B–F) two‐tailed Student's unpaired *t*‐test. (B, C, E) ***P < 0.0001, (D) *P = 0.0473, (F) **P = 0.0015.

CX_3_CL1 expression after CI prompted us to investigate leukocyte recruitment in *Cx3cr1* ^*GFP/+*^ reporter mice, in which green fluorescent protein (GFP) is expressed in different intensities in Ly6C^hi^ (GFP^lo^) and Ly6C^lo^ (GFP^hi^) monocyte subsets (Jung *et al*, 2000; Auffray *et al*, 2007). In confocal microscopy, recruitment kinetics of GFP^+^ cells to the EC wound mirrored that of CX_3_CL1 expression and EC regeneration: a static phase of minimal recruitment on day 2, followed by a significant and progressive increase during the dynamic phase (Fig 2A and B). Furthermore, analysis of GFP intensities with different amplifier gain settings (Auffray *et al*, 2007) revealed that recruited GFP^+^ cells were GFP^hi^, suggesting recruitment of Ly6C^lo^ monocytes (Figs 2C and EV1A). Additional *in situ* analysis revealed expression of CD11b but negative staining for NK1.1 or CD90.2, findings consistent with recruitment of Ly6C^lo^ monocytes, but not NK or T cells (Fig EV1B). Furthermore, proliferation in the wound edge was restricted to EC and did not involve GFP^+^ cells, suggesting progressive recruitment of GFP^+^ cells during wound closure (Fig EV1C). Also, no GFP expression was observed in resident EC (Figs 2A and D, and 3A).

To investigate whether Ly6C^lo^ monocytes are recruited to the endothelial monolayer, we scanned the entire dimension of the vessel wall along the z‐axis by confocal microscopy and performed 3D reconstruction of the z‐stack. This revealed that Ly6C^lo^ monocytes are found only within the EC monolayer near the wound edge (Fig 2D).

**Figure EV2:**
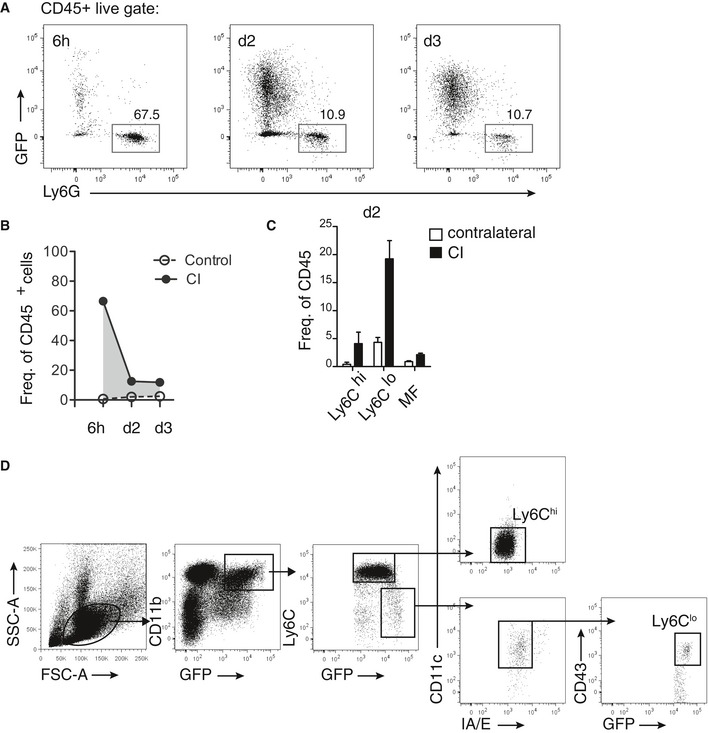
Analysis of myeloid infiltration after carotid injury by flow cytometry Representative flow cytometry plot depicting granulocyte (CD11b^+^GFP^−^Ly6G^+^) infiltration 6 h, d2 and d3 after carotid injury.Quantitative analysis of myeloid cell infiltration depicted as frequency of CD45^+^PI^−^ (live) cells showing specific infiltration of Ly6C^hi^, Ly6C^lo^, and macrophages of injury site but not of contralateral uninjured carotid artery with CD45^+^ cells. 6 h *n* = 3, d2 *n* = 5, d3 *n* = 3.Quantification of myeloid infiltration displayed as frequency of live cells showing that infiltration is specific to injured carotid at d2.Sorting strategy employed for isolation of Ly6C^hi^ (blue) and Ly6C^lo^ (black) murine monocyte subsets from bone marrow.Data information: In (B, C), data are presented as mean ± SEM.

To more precisely characterize the phenotype and recruitment kinetics of leukocyte populations, we performed serial flow cytometric analysis from carotids of *Cx3cr1* ^*GFP/+*^ mice (Fig 2E; Galkina *et al*, 2006; Gamrekelashvili *et al*, 2016). Neutrophils were recruited early after carotid injury (6 h) and declined rapidly thereafter (Fig EV2A and B). Recruitment of Ly6C^hi^ monocytes and macrophages remained steady at low levels. In contrast, homing of Ly6C^lo^ monocytes, defined as Ly6C^lo^/MHC II^neg^/CD11c^+^/GFP^hi^ (see Appendix Table S3), progressively increased in injured carotids over time (Figs 2E and F, and EV2C). Thus, our data demonstrate that the dynamic phase of endothelial regeneration is characterized by coordinate induction of CX_3_CL1 and recruitment of Ly6C^lo^ monocytes to the injured endothelium.

### Monocyte subsets regulate EC proliferation and regeneration

To further investigate the relevance of CX_3_CL1‐mediated monocyte recruitment for EC regeneration *in vivo,* we studied mice with *Cx3cr1* loss of function (*Cx3cr1* ^*GFP/GFP*^), which have normal circulating monocyte levels but defective Ly6C^lo^ monocytes recruitment to endothelial CX_3_CL1 (Carlin *et al*, 2013). Following CI, recruitment of GFP^+^ cells was strongly impaired in *Cx3cr1* mutant mice (Fig 3A and B). Impaired monocyte recruitment in *Cx3cr1*‐deficient mice was accompanied by reduced EC proliferation (Fig 3C). Furthermore, the remaining EC wound area was markedly larger in *Cx3cr1*‐deficient mice compared to controls, demonstrating defective EC regeneration after arterial injury in mice with *Cx3cr1* loss of function (Fig 3D). Importantly, these defects were rescued by adoptive transfer of sorted Ly6C^lo^ monocytes from *Cx3cr1* ^GFP/+^ donor mice, which increased the number of GFP^+^ monocytes at the wound site (Fig 3E) and promoted endothelial wound closure after CI (Fig 3F).

**Figure EV3:**
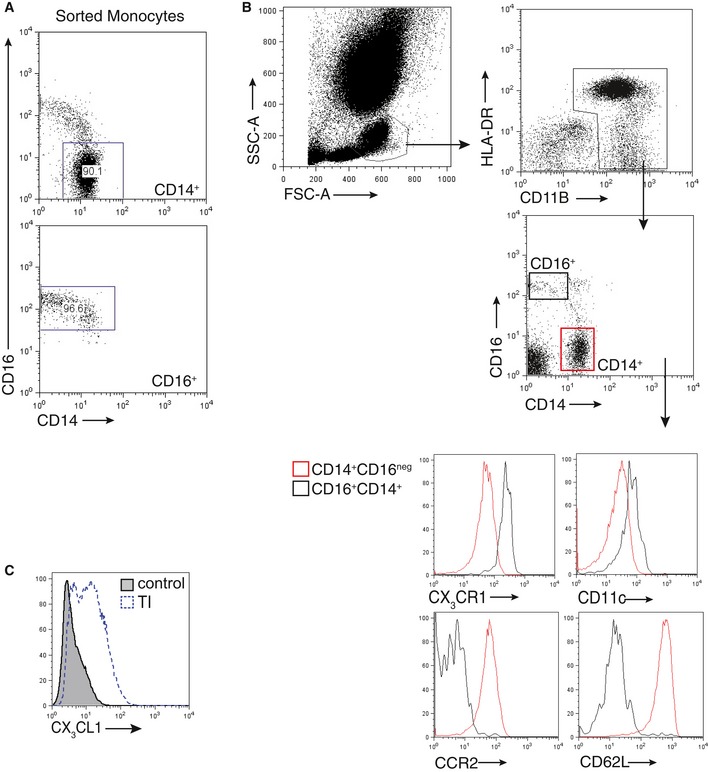
Profile and isolation of human monocyte subsets and CX_3_ CL1 expression by EC Analysis of CD14^++^CD16^neg^ (CD14^+^) and CD14^+^CD16^++^ (CD16^+^) sorted monocytes after magnetic bead based sorting. Purity levels of > 90% for both sorted monocyte subsets.Gating strategy for human monocyte subset characterization from PBMC (upper panel) and representative expression profiles from monocyte subsets (bottom) by flow cytometry.Representative histogram depicting CX_3_CL1 induction upon stimulation of HAECs with recombinant human TNF‐α/IFN‐γ (open dotted line). Representative histogram with unstimulated HAECs as a control (gray, shaded) is shown.

**Figure 4:**
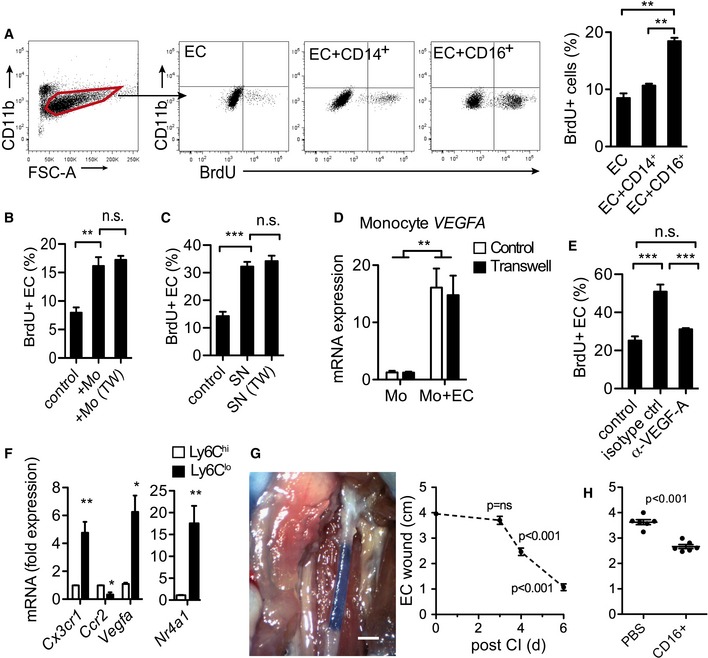
Monocytes induce EC proliferation and regeneration Representative flow cytometry analysis of EC BrdU incorporation after co‐culture with human monocytes (left) and quantification of EC proliferation (right). *n* = 3.EC proliferation after co‐culture with non‐classical monocytes with or without Transwell insert (TW), *n* = 3.EC proliferation with conditioned medium (SN) from co‐cultures generated with or without Transwell insert (TW), *n* = 3.Gene expression by quantitative RT–PCR from non‐classical monocytes cultured with or without EC for 24 h, with or without Transwell insert, normalized to input monocytes. *n* = 3.EC proliferation after 3 days co‐culture with non‐classical monocytes with VEGFA neutralizing antibody or isotype control, *n* = 3.Quantitative RT–PCR of murine monocyte subsets, normalized to gene expression of Ly6C^hi^ monocyte expression levels, *n* = 3.Representative image (left) and quantification of EC wound closure (right) after CI in nude mice analyzed with Evans blue staining. Scale bar: 1 cm, *n* = 3 per group.Quantification of wound closure after CI and transfer of non‐classical monocytes (5 × 10^5^cells/mouse). *n* = 6/6.Data information: Data are presented as mean ± SEM. Statistical analysis: (A–C, E, G) one‐way ANOVA with Bonferroni's multiple‐comparison test; (D) two‐way ANOVA with Bonferroni's multiple‐comparison test; ***P < 0.001, **P < 0.01, *P < 0.05. (F, H) Two‐tailed unpaired Student's *t*‐test, *Cx3cr1*: **P = 0.0014, *Ccr2*: *P = 0.0254, *Vegfa*: *P = 0.0238, *Nr4a1*: **P = 0.0017.

To test whether monocyte subsets regulate EC proliferation, we isolated human classical (CD14^++^CD16^neg^) and non‐classical (CD14^+^CD16^++^) monocytes from peripheral blood (Fig EV3A), which show conserved phenotypic and functional characteristics including CX_3_CR1 expression (Fig EV3B; Ingersoll *et al*, 2010), and co‐cultured them with human aortic EC (HAEC) pretreated with TNF‐α/IFN‐γ to induce CX_3_CL1 (Fig EV3C; Schulz *et al*, 2007). While classical monocytes did not induce EC proliferation to a significant extend, non‐classical monocytes approximately doubled the EC proliferation rate, which also occurred when direct cell contact was prevented by a Transwell insert (Fig 4A and B). In addition, EC proliferation was induced by addition of medium conditioned with supernatants from co‐culture experiments (Fig 4C), which together suggested a secreted factor mediating EC proliferation. Indeed, monocytes cultured in the presence of EC showed increased levels of vascular endothelial growth factor A (VEGFA) irrespective of EC‐contact (Fig 4D). Importantly, addition of a VEGFA neutralizing antibody abrogated induction of EC proliferation by monocytes (Fig 4E). Furthermore, increased *Vegfa* expression was also confirmed in bona fide Ly6C^lo^ monocytes compared to Ly6C^hi^ monocytes (Fig 4F).

To test the capacity of human non‐classical monocytes to promote EC regeneration, we performed a hybrid adoptive transfer experiment in which human monocytes are transferred into immunocompromised nude mice after CI. The effect on endothelial wound healing was measured by Evans blue injection, which stains the de‐endothelialized region intensely and uniformly blue (Carmeliet *et al*, 1997; Sorrentino *et al*, 2007). Interestingly, also in the nude mouse model, the principle separation into a static and dynamic phase during endogenous EC regeneration was conserved, the dynamic phase starting after d3 postinjury (Fig 4G). Importantly, adoptive transfer of human non‐classical monocytes improved endothelial wound closure during EC regeneration (Fig 4H). These results demonstrate that patrolling monocytes promote endothelial regeneration in the arterial circulation after injury.

## Discussion

Endothelial cells are quiescent in normal arteries but start to proliferate and replicate after vascular injury, which is required for re‐endothelialization of larger endothelial wounds to prevent neointima formation or clotting (Schwartz *et al*, 1978; Haudenschild & Schwartz, 1979; Reidy & Schwartz, 1981).

We here show that the transition from a static phase to a dynamic phase of re‐endothelialization is characterized by strong induction of endothelial CX_3_CL1 expression and consecutive recruitment of *Cx3cr1‐*expressing Ly6C^lo^ monocytes into the endothelial monolayer. Notably, endothelial CX_3_CL1 induction was also observed in a related vascular wire injury model (Liu *et al*, 2006). CX_3_CL1 is a unique ligand for CX_3_CR1 and acts as an adhesion molecule that promotes the firm adhesion of CX_3_CR1‐expressing monocyte subsets to EC (Fong *et al*, 1998; Ancuta *et al*, 2003). Indeed, the membrane‐bound form of CX_3_CL1 is predestined to serve as a homing cue under physiological flow, since it captures leukocytes without requiring selectin‐mediated rolling or activation of integrins, while secreted chemokines are carried away with the blood stream (Bazan *et al*, 1997; Imai *et al*, 1997). Currently, the regulation of CX_3_CL1 expression *in vivo* is unknown, but might involve inflammatory stimuli, such as TNF‐α, IFN‐γ, or TLR7 activation, which are involved in the vascular injury response and upregulate CX_3_CL1 *in vitro* and *in vivo* (Zimmerman *et al*, 2002; Ahn *et al*, 2004; Schulz *et al*, 2007; Carlin *et al*, 2013).

Our unbiased analysis of myeloid cell subsets revealed that the immediate response to endothelial injury is dominated by neutrophils, which is in line with previous reports (Welt *et al*, 2000). The dynamic phase of EC regeneration, however, was characterized by recruitment of *Cx3cr1*‐expressing Ly6C^lo^ monocytes, identified by flow cytometry and *in situ* confocal imaging based on prototypical profiles (Ingersoll *et al*, 2010; Gamrekelashvili *et al*, 2016; Krishnasamy *et al*, 2017). Several lines of evidence suggest that Ly6C^lo^ monocytes and their human counterparts, non‐classical monocytes, are critically involved in arterial EC wound healing after injury. Recruitment of Ly6C^lo^ monocytes steadily and significantly increased during the course of endothelial wound healing, coinciding with the onset of proliferation, while Ly6C^hi^ monocyte recruitment remained constant, but on a much lower level. Both mouse and human monocyte subsets express high levels of CX_3_CR1, which mediates firm attachment to CX_3_CL1‐expressing EC after injury (Carlin *et al*, 2013), and endothelial wound healing was impaired in *Cx3cr1*‐deficient mice that have normal circulating levels of monocytes subsets (Auffray *et al*, 2009). Ly6C^lo^ monocytes promote EC wound healing by recruiting neutrophils to mediate clearance of injured EC (Carlin *et al*, 2013) or by secreting growth factors like VEGF (Nahrendorf *et al*, 2007). Indeed, we show that mouse and human patrolling monocyte subsets express VEGF, which is required for induction of EC proliferation *in vitro*. Finally, human non‐classical human monocytes improved EC wound closure in an adoptive transfer model, indicating that the regenerative potential of patrolling monocytes is conserved, which warrants further investigations as cell‐based therapeutics to repair endothelial wounds in damaged arteries.

## Materials and Methods

Additional materials and methods are listed in the Appendix.

### Animals and surgical procedures

*Cx3cr1* ^*GFP/+*^ reporter mice have been described previously (Jung *et al*, 2000). Mice were housed under specific pathogen‐free conditions, and age‐ and sex‐matched wild‐type C57BL/6J mice or littermates were used as controls. Animal experiments were approved by the local animal welfare board. Carotid perivascular electric injury was performed with the following modification as previously described (Carmeliet *et al*, 1997; Sorrentino *et al*, 2007). Ten‐ to 15‐week‐old male mice were anaesthetized with a mixture of ketamine (80 mg/kg), xylazine (2.5 mg/kg), and midazolam (2.5 mg/kg) injected i.p., and the fur of the neck area was shaved completely. Bepanthen eye cream (Bayer) was applied to the eyes to prevent desiccation and unnecessary damage. In order to avoid hypothermia, the animals were placed on a heating pad and kept at a constant 37°C. An opening incision of 1–2 cm was placed along the midline of the neck, and a small portion of the left distal common carotid artery was laid free. Endothelial injury was induced with bipolar forceps (Cat. 20195‐066, Erbe Elektromedizin GmbH) placed on the blood vessel. An injury was made through an electric impulse applied (intensity 2 W, bipolar mode) once for 2 s using Erbe microregulator (Erbotom ICC 50). The incision was sutured using polyester thread (polyester‐S, 2xDRT 12, 6/0 USP, Catgut GmbH).

### Human endothelial cell culture and monocyte isolation

The experiments conform to the principles set out in the WMA Declaration of Helsinki and the Department of Health and Human Services Belmont Report and were approved by the local ethics board. Human CD14^++^CD16^neg^ and CD14^+^CD16^++^ monocytes were isolated from blood rings of healthy donors after written consent (Blood bank, Medizinische Hochschule Hannover) using CD14 microbeads and CD16 monocyte isolation kit (Miltenyi Biotec), respectively, according to manufacturer's instructions. The purity of the isolated cells was more than 90% routinely tested by flow cytometry. Human aortic endothelial cells (HAEC) were purchased from Lonza and cultured as per manufacturer's instructions.

### Statistical analysis

All data are presented as mean ± SEM. Significance of differences was calculated using unpaired, two‐tailed Student's *t‐*test. For comparison of multiple experimental groups, one‐way ANOVA and Bonferroni's multiple‐comparison test were used. *P*‐values of less than 0.05 were considered to be significant and are indicated by the asterisk (***P < 0.001, **P < 0.01, *P < 0.05).

## Author contributions

TG, KK, AL, TK, CH, and LCN performed experiments. TG, KK, JG, AL, CH, and FPL analyzed the data. KK, TG, JG, JB, HH, and FPL wrote and edited the manuscript. FPL conceived the study.

## Conflict of interest

The authors declare that they have no conflict of interest.

## Supporting information



Appendix

Expanded View Figures PDF

Review Process File

## References

[emmm201607502-bib-0001] Ahn SY , Cho CH , Park KG , Lee HJ , Lee S , Park SK , Lee IK , Koh GY (2004) Tumor necrosis factor‐alpha induces fractalkine expression preferentially in arterial endothelial cells and mithramycin A suppresses TNF‐alpha‐induced fractalkine expression. Am J Pathol 164: 1663–1672 15111313 10.1016/s0002-9440(10)63725-x PMC1615656

[emmm201607502-bib-0002] Akimoto S , Mitsumata M , Sasaguri T , Yoshida Y (2000) Laminar shear stress inhibits vascular endothelial cell proliferation by inducing cyclin‐dependent kinase inhibitor p21(Sdi1/Cip1/Waf1). Circ Res 86: 185–190 10666414 10.1161/01.res.86.2.185

[emmm201607502-bib-0003] Ancuta P , Rao R , Moses A , Mehle A , Shaw SK , Luscinskas FW , Gabuzda D (2003) Fractalkine preferentially mediates arrest and migration of CD16^+^ monocytes. J Exp Med 197: 1701–1707 12810688 10.1084/jem.20022156 PMC2193954

[emmm201607502-bib-0004] Auffray C , Fogg D , Garfa M , Elain G , Join‐Lambert O , Kayal S , Sarnacki S , Cumano A , Lauvau G , Geissmann F (2007) Monitoring of blood vessels and tissues by a population of monocytes with patrolling behavior. Science 317: 666–670 17673663 10.1126/science.1142883

[emmm201607502-bib-0005] Auffray C , Fogg DK , Narni‐Mancinelli E , Senechal B , Trouillet C , Saederup N , Leemput J , Bigot K , Campisi L , Abitbol M *et al* (2009) CX3CR1^+^ CD115^+^ CD135^+^ common macrophage/DC precursors and the role of CX3CR1 in their response to inflammation. J Exp Med 206: 595–606 19273628 10.1084/jem.20081385 PMC2699130

[emmm201607502-bib-0006] Bazan JF , Bacon KB , Hardiman G , Wang W , Soo K , Rossi D , Greaves DR , Zlotnik A , Schall TJ (1997) A new class of membrane‐bound chemokine with a CX3C motif. Nature 385: 640–644 9024663 10.1038/385640a0

[emmm201607502-bib-0007] Becher UM , Moller L , Tiyerili V , Vasa Nicotera M , Hauptmann F , Zimmermann K , Pfeifer A , Nickenig G , Wassmann S , Werner N (2014) Distinct CD11b^+^‐monocyte subsets accelerate endothelial cell recovery after acute and chronic endothelial cell damage. Int J Cardiol 173: 80–91 24602320 10.1016/j.ijcard.2014.02.004

[emmm201607502-bib-0008] Carlin LM , Stamatiades EG , Auffray C , Hanna RN , Glover L , Vizcay‐Barrena G , Hedrick CC , Cook HT , Diebold S , Geissmann F (2013) Nr4a1‐dependent Ly6C(low) monocytes monitor endothelial cells and orchestrate their disposal. Cell 153: 362–375 23582326 10.1016/j.cell.2013.03.010 PMC3898614

[emmm201607502-bib-0009] Carmeliet P , Moons L , Stassen JM , De Mol M , Bouche A , van den Oord JJ , Kockx M , Collen D (1997) Vascular wound healing and neointima formation induced by perivascular electric injury in mice. Am J Pathol 150: 761–776 9033288 PMC1858279

[emmm201607502-bib-0010] Chen D , Walsh K , Wang J (2000) Regulation of cdk2 activity in endothelial cells that are inhibited from growth by cell contact. Arterioscler Thromb Vasc Biol 20: 629–635 10712384 10.1161/01.atv.20.3.629

[emmm201607502-bib-0011] Cros J , Cagnard N , Woollard K , Patey N , Zhang S‐Y , Senechal B , Puel A , Biswas SK , Moshous D , Picard C *et al* (2010) Human CD14dim monocytes patrol and sense nucleic acids and viruses via TLR7 and TLR8 receptors. Immunity 33: 375–386 20832340 10.1016/j.immuni.2010.08.012 PMC3063338

[emmm201607502-bib-0012] Filipe C , Lam Shang Leen L , Brouchet L , Billon A , Benouaich V , Fontaine V , Gourdy P , Lenfant F , Arnal JF , Gadeau AP *et al* (2008) Estradiol accelerates endothelial healing through the retrograde commitment of uninjured endothelium. Am J Physiol Heart Circ Physiol 294: H2822–H2830 18441207 10.1152/ajpheart.00129.2008

[emmm201607502-bib-0013] Flierl U , Bauersachs J , Schafer A (2015) Modulation of platelet and monocyte function by the chemokine fractalkine (CX3 CL1) in cardiovascular disease. Eur J Clin Invest 45: 624–633 25832902 10.1111/eci.12443

[emmm201607502-bib-0014] Fong AM , Robinson LA , Steeber DA , Tedder TF , Yoshie O , Imai T , Patel DD (1998) Fractalkine and CX3CR1 mediate a novel mechanism of leukocyte capture, firm adhesion, and activation under physiologic flow. J Exp Med 188: 1413–1419 9782118 10.1084/jem.188.8.1413 PMC2213407

[emmm201607502-bib-0015] Galkina E , Kadl A , Sanders J , Varughese D , Sarembock IJ , Ley K (2006) Lymphocyte recruitment into the aortic wall before and during development of atherosclerosis is partially L‐selectin dependent. J Exp Med 203: 1273–1282 16682495 10.1084/jem.20052205 PMC2121208

[emmm201607502-bib-0016] Gamrekelashvili J , Giagnorio R , Jussofie J , Soehnlein O , Duchene J , Briseno CG , Ramasamy SK , Krishnasamy K , Limbourg A , Kapanadze T *et al* (2016) Regulation of monocyte cell fate by blood vessels mediated by Notch signalling. Nat Commun 7: 12597 27576369 10.1038/ncomms12597 PMC5013671

[emmm201607502-bib-0017] Gimbrone MA Jr , Garcia‐Cardena G (2016) Endothelial cell dysfunction and the pathobiology of atherosclerosis. Circ Res 118: 620–636 26892962 10.1161/CIRCRESAHA.115.306301 PMC4762052

[emmm201607502-bib-0018] Haudenschild CC , Schwartz SM (1979) Endothelial regeneration. II. Restitution of endothelial continuity. Lab Invest 41: 407–418 502473

[emmm201607502-bib-0019] Imai T , Hieshima K , Haskell C , Baba M , Nagira M , Nishimura M , Kakizaki M , Takagi S , Nomiyama H , Schall TJ *et al* (1997) Identification and molecular characterization of fractalkine receptor CX3CR1, which mediates both leukocyte migration and adhesion. Cell 91: 521–530 9390561 10.1016/s0092-8674(00)80438-9

[emmm201607502-bib-0020] Ingersoll MA , Spanbroek R , Lottaz C , Gautier EL , Frankenberger M , Hoffmann R , Lang R , Haniffa M , Collin M , Tacke F *et al* (2010) Comparison of gene expression profiles between human and mouse monocyte subsets. Blood 115: e10–e19 19965649 10.1182/blood-2009-07-235028 PMC2810986

[emmm201607502-bib-0021] Jung S , Aliberti J , Graemmel P , Sunshine MJ , Kreutzberg GW , Sher A , Littman DR (2000) Analysis of fractalkine receptor CX(3)CR1 function by targeted deletion and green fluorescent protein reporter gene insertion. Mol Cell Biol 20: 4106–4114 10805752 10.1128/mcb.20.11.4106-4114.2000 PMC85780

[emmm201607502-bib-0022] Koenen RR , Weber C (2011) Chemokines: established and novel targets in atherosclerosis. EMBO Mol Med 3: 713–725 22038924 10.1002/emmm.201100183 PMC3377113

[emmm201607502-bib-0023] Krishnasamy K , Limbourg A , Kapanadze T , Gamrekelashvili J , Beger C , Häger C , Lozanovski VJ , Falk CS , Napp LC , Bauersachs J *et al* (2017) Blood vessel control of macrophage maturation promotes arteriogenesis in ischemia. Nat Commun 8: 952 29038527 10.1038/s41467-017-00953-2 PMC5643305

[emmm201607502-bib-0024] Liu P , Patil S , Rojas M , Fong AM , Smyth SS , Patel DD (2006) CX3CR1 deficiency confers protection from intimal hyperplasia after arterial injury. Arterioscler Thromb Vasc Biol 26: 2056–2062 16809547 10.1161/01.ATV.0000234947.47788.8c

[emmm201607502-bib-0025] Nahrendorf M , Swirski FK , Aikawa E , Stangenberg L , Wurdinger T , Figueiredo JL , Libby P , Weissleder R , Pittet MJ (2007) The healing myocardium sequentially mobilizes two monocyte subsets with divergent and complementary functions. J Exp Med 204: 3037–3047 18025128 10.1084/jem.20070885 PMC2118517

[emmm201607502-bib-0026] Quintar A , McArdle S , Wolf D , Marki A , Ehinger E , Vassallo M , Miller J , Mikulski Z , Ley K , Buscher K (2017) Endothelial protective monocyte patrolling in large arteries intensified by western diet and atherosclerosisnovelty and significance. Circ Res 120: 1789–1799 28302649 10.1161/CIRCRESAHA.117.310739 PMC5446289

[emmm201607502-bib-0027] Reidy MA , Schwartz SM (1981) Endothelial regeneration. III. Time course of intimal changes after small defined injury to rat aortic endothelium. Lab Invest 44: 301–308 7206628

[emmm201607502-bib-0028] Schafer A , Schulz C , Eigenthaler M , Fraccarollo D , Kobsar A , Gawaz M , Ertl G , Walter U , Bauersachs J (2004) Novel role of the membrane‐bound chemokine fractalkine in platelet activation and adhesion. Blood 103: 407–412 12969973 10.1182/blood-2002-10-3260

[emmm201607502-bib-0029] Schubert SY , Benarroch A , Ostvang J , Edelman ER (2008) Regulation of endothelial cell proliferation by primary monocytes. Arterioscler Thromb Vasc Biol 28: 97–104 17991870 10.1161/ATVBAHA.107.157537

[emmm201607502-bib-0030] Schulz C , Schafer A , Stolla M , Kerstan S , Lorenz M , von Bruhl ML , Schiemann M , Bauersachs J , Gloe T , Busch DH *et al* (2007) Chemokine fractalkine mediates leukocyte recruitment to inflammatory endothelial cells in flowing whole blood: a critical role for P‐selectin expressed on activated platelets. Circulation 116: 764–773 17679613 10.1161/CIRCULATIONAHA.107.695189

[emmm201607502-bib-0031] Schwartz SM , Haudenschild CC , Eddy EM (1978) Endothelial regeneration. I. Quantitative analysis of initial stages of endothelial regeneration in rat aortic intima. Lab Invest 38: 568–580 642457

[emmm201607502-bib-0032] Sorrentino SA , Bahlmann FH , Besler C , Muller M , Schulz S , Kirchhoff N , Doerries C , Horvath T , Limbourg A , Limbourg F *et al* (2007) Oxidant stress impairs *in vivo* reendothelialization capacity of endothelial progenitor cells from patients with type 2 diabetes mellitus: restoration by the peroxisome proliferator‐activated receptor‐gamma agonist rosiglitazone. Circulation 116: 163–173 17592079 10.1161/CIRCULATIONAHA.106.684381

[emmm201607502-bib-0033] Welt FG , Edelman ER , Simon DI , Rogers C (2000) Neutrophil, not macrophage, infiltration precedes neointimal thickening in balloon‐injured arteries. Arterioscler Thromb Vasc Biol 20: 2553–2558 11116052 10.1161/01.atv.20.12.2553

[emmm201607502-bib-0034] Yona S , Kim KW , Wolf Y , Mildner A , Varol D , Breker M , Strauss‐Ayali D , Viukov S , Guilliams M , Misharin A *et al* (2013) Fate mapping reveals origins and dynamics of monocytes and tissue macrophages under homeostasis. Immunity 38: 79–91 23273845 10.1016/j.immuni.2012.12.001 PMC3908543

[emmm201607502-bib-0035] Zimmerman MA , Selzman CH , Reznikov LL , Miller SA , Raeburn CD , Emmick J , Meng X , Harken AH (2002) Lack of TNF‐alpha attenuates intimal hyperplasia after mouse carotid artery injury. Am J Physiol Regul Integr Comp Physiol 283: R505–R512 12121864 10.1152/ajpregu.00033.2002

