# Supplementary material for: The chemokine receptor CX 3 CR1 coordinates monocyte recruitment and endothelial regeneration after arterial injury
Source: EMBO Mol Med. 2017 Dec 11;10(2):151–9. doi: 10.15252/emmm.201707502 (PMC5801509; doi:10.15252/emmm.201707502)
Supplement: Supplementary file 1 — Appendix [file EMMM-10-151-s001.pdf]

Appendix

The chemokine receptor CX3CR1 coordinates monocyte recruitment and endothelial regeneration after arterial injury

Tobias Getzin[1,4,#], Kashyap Krishnasamy[1,2,#], Jaba Gamrekelashvili[1,2], Tamar Kapanadze[1,2], Anne Limbourg[1,5], Christine Häger[1,6], L. Christian Napp[1,3], Johann Bauersachs[3], Hermann Haller[2], Florian P. Limbourg[1,2]

1 Vascular Medicine Research, Hannover Medical School, Hannover, Germany
2 Department of Nephrology and Hypertension, Hannover Medical School, Hannover, Germany
3 Department of Cardiology and Angiology, Hannover Medical School, Hannover, Germany
4 Current address: Institute of Radiology, Hannover Medical School, Hannover, Germany
5 Current address: Department of Plastic, Aesthetic, Hand and Reconstructive Surgery, Hannover Medical School, Hannover, Germany
6 Current address: Institute for Laboratory Animal Science and Central Animal Facility, Hannover Medical School, Hannover, Germany
# equal contribution

Table of contents

Appendix methods	02-05
Appendix references for methods	05
Appendix table S1	06
Appendix table S2	07
Appendix table S3	08
Appendix table S4	09
Appendix table S5	10

Appendix methods

Tissue preparation and analysis

Mice were anesthetized and perfused by injecting fixation solutions sequentially into the left ventricle of the beating heart: PBS (with Ca^{2+} and Mg^{2+}, Lonza), 4% PFA (Sigma-Aldrich) with 0.1% Glutaraldehyde (Grade I, Sigma-Aldrich), 4% PFA with 0.1% GA and 10% Sucrose (D (+)-Saccharose, Carl Roth) and finally 20% Sucrose. Common carotid arteries were extracted using microsurgical scissors and a Zeiss Stemi DV4 Sport surgical microscope by separating the internal and external carotid artery just above the bifurcation. Special care was taken not to handle or stress the injury site. To ensure proper staining with antibodies the arteries were cut open and fixed longitudinally under a Zeiss Stemi 2000-C stereo microscope, using microsurgical scissors and forceps. For Flow cytometry analysis mice were euthanized and injured, contralateral carotid arteries were excised without fixation. Tissue was digested using Collagenase Type 2 (Worthington Biochemical) and single cell suspension was prepared.

Immunohistochemistry, Confocal laser scanning microscopy and image processing

The carotid arteries were placed on microscopic slides. Nonspecific binding of antibodies was prevented using blocking buffer (0.3% Triton X-100, 5% normal serum in PBS) at room temperature for 4 hours. After blocking slides were stained with the primary antibody (4°C, O/N) followed by washing and overnight staining steps with secondary antibody conjugates (4°C) described in appendix table S1. Finally, tissue slides were washed, counterstained with DAPI and mounted with tissue mounting medium (Fluorescent mounting medium, DAKO). Samples were imaged with

fluorescent microscope (Observer Z1, Carl Zeiss AG) or confocal microscope (Leica DM IRB2, Leica microsystems).

Confocal microscopy was performed using a Leica DM IRB2 microscope equipped with TCS SP2 AOBS scan head and 40x and 63x Oil immersion objectives. To characterise monocyte subsets based on GFP expression confocal images were acquired using different amplification levels of green detector (amplifier gain) which allows to distinguish between GFP^{hi} and GFP^{lo} cells as described previously(Auffray et al, 2007). Amplifier gain for green detector was set to 600V (Low amplifier gain) to identify GFP^{hi} cells. After acquisition of the image amplifier gain was set to 800V (High amplifier gain) and the same area was imaged again to detect cells expressing low levels of GFP. Leica confocal software (LCS Lite) was used to create Z-stacks of confocal images, which were later merged together for 3D reconstruction.

Human endothelial cell culture and monocyte isolation

Human $CD14^{++}CD16^{neg}$ and $CD14^{+}CD16^{++}$ monocytes were isolated from blood rings of healthy donors (Blood bank, Medizinische Hochschule Hannover) using CD14 microbeads and CD16 monocyte isolation kit (Mitenyi Biotec) respectively according to manufacturer's instructions. The purity of the isolated cells was more than 90% routinely tested by flow cytometry. Human Aortic Endothelial cells (HAEC) were purchased from Lonza and cultured as per manufacturer's instructions.

***in vitro* co-culture studies**

For proliferation assay, semi-confluent HAECs were serum starved for 12-16 hours, stimulated with recombinant human TNF-α (25μg/ml, Peprotech) and recombinant human IFN-γ (20μg/ml, Peprotech) to induce CX_3CL1 expression and co-cultured with $CD14^{++}CD16^{neg}$ or $CD14^{+}CD16^{++}$ human monocytes. 24 hours after incubation

10μM BrdU was applied to the cultures and incorporation of it in EC was measured by flow cytometry.

For proliferation assays cells were incubated with 10μM BrdU for 12 hours. Cultured cells were collected, stained using BrdU flow kit (BD Pharmingen) according to manufacturer's instructions and analyzed by flow cytometry.

For the Transwell assay, Monocytes and EC's were cultured separated by a transwell (Polycarbonate membrane,12mm diameter inserts, 0.4μm pore size from Corning Inc) for 48 hours. For VEGF neutralization assays, EC's were pre-treated with anti-human VEGF neutralizing antibody (100ng/mL, Affinity purified goat IgG, Catalog no: AF-293-NA, R&D systems) for the duration of the co-culture (48 hrs). For conditioned medium experiments, supernatants from 48hr co-cultures were harvested, spun at 12000 xg to remove cell debris and diluted 1:1 with EBM2+0.5% FCS. The diluted supernatants were used for the conditioned medium experiments.

Flow cytometry

After blocking of non-specific binding with anti-mouse CD16/32 (TruStain fcX, Biolegend) cells were stained with appropriate primary and secondary antibodies or streptavidin-fluorochrome conjugates (appendix table S2) and used for flow cytometry analysis (LSR-II, BD Biosciences) Data were analysed by FlowJo software (Treestar). Initially cells were identified based on FSC and SSC characteristics. After exclusion of doublets (on the basis of SSC-W, SSC-A), frequency of each subpopulation from 7-AAD negative (live) cell gate was determined and is shown in the graphs as mean±s.e.m, unless otherwise stated.

RNA analysis by real-time quantitative PCR

RNA was isolated with the Nucleospin II kit (Macherey Nagel) and Reverse transcription was done using Reverse transcriptase Kit (Invitrogen), according to manufacturer's protocols. For gene expression analysis, primers were designed using the software Primerquest (IDT) and PrimerBLAST (NCBI) following general guidelines for primer design. Quantitative RT-PCR analysis was performed in duplicates for each sample using Fast start essential green DNA master mix (Roche GmbH) according to manufacturer's instructions with murine/human primers listed in appendix table S4, S5 on Light cycler 96 system (Roche). Murine *Rps9* was used as a housekeeping gene to normalize expression of gene of interest depending on the experiment. Relative expression was calculated by the comparative CT ($2^{-\Delta\Delta Ct}$ values indicate fold change of the gene of interest in the samples relative to a selected control).

References for methods

Auffray C, Fogg D, Garfa M, Elain G, Join-Lambert O, Kayal S, Sarnacki S, Cumano A, Lauvau G, Geissmann F (2007) Monitoring of blood vessels and tissues by a population of monocytes with patrolling behavior. Science 317**:** 666-670

Appendix table S1.

Antibodies for flow cytometry used in the study

Antibody/dye	Clone	Usage
Anti-mouse CD45	30-F11	1:400
Anti-mouse CD11b	M1/70	1:400
Anti-mouse Ly-6C	HK1.4	1:1200
Anti-mouse MHC-II	M5/114.15.2	1:100
Anti-mouse CD11c	N418	1:100
Anti-mouse F4/80	BM8	1:100
Anti-mouse Ly-6G	1A8	1:400
7-AAD		
BrdU		
Anti-human CD14	MφP9	1:100
Anti-human CD16	3G8	1:100
Anti-human CD11b	ICRF44	1:400
Anti-human CX3CL1	51637	1:50
Anti-human CX3CR1	2A9-1	1:100
DAPI		1:1000

Appendix table S2.

Antibodies and secondary conjugates used for Immunohistochemistry

Antibody/dye	Clone	Stock Concentration	Usage
Purified Rat anti-mouse CD31	MEC 13.3	15.625µg/ml	1:100
Rabbit polyclonal CX3CL1		1mg/ml	1:100
Purified Rat anti-mouse CD11b	M 1/70	500µg/ml	1:100
Biotin Hamster anti-mouse CD11c	HL3	500µg/ml	1:100
Purified Rat anti-mouse CD45	30-F11	62.5µg/ml	1:100
Monoclonal Rat anti-mouse Ki-67	TEC-3	7.4g/L	
Biotin Rat anti-mouse Ly6C	AL-21	500µg/ml	1:100
Purified Rabbit anti-GFP	SP3005	1mg/ml	1:100
CY3 Mouse anti-biotin		1.7mg/ml	1:100
PE Rat anti-mouse CD90.2	53-2.1	200µg/ml	1:100
Cy™3-conjugated* AffiniPure Donkey anti-rat IgG (H+L)	3G8	1.5mg/dl	1:100
Cy™3-conjugated* AffiniPure Goat anti-rabbit IgG (H+L)	ICRF44	1.5mg/ml	1:100
PE Mouse anti-mouse NK1.1	PK136	200µg/ml	1:100

Appendix table S3.

Phenotypic characterization and gating strategy for flow cytometry

Cell type	Phenotype
Granulocytes	$CD45^{+}CD11b^{+}GFP^{neg}Ly6G^{+}$
$Ly6C^{hi}$ monocytes	$CD45^{+}CD11b^{+}MHC\text{-}II^{neg}CD11c^{neg}Ly6C^{hi}GFP^{lo}$
$Ly6C^{lo}$ monocytes	$CD45^{+}CD11b^{+}MHC\text{-}II^{neg}CD11c^{+}Ly6C^{lo}GFP^{hi}$
Macrophages	$CD45^{+}CD11b^{+}MHC\text{-}II^{+}Ly6C^{lo}GFP^{lo}$

Appendix table S4.

List of murine real time PCR primers

Gene	Primer pair
Rps9	Forward: GGA TTT CTT GGA GAG GCG GC<br>Reverse: ACC TGC TTG CGG ACC CTA AT
Cx3cr1	Forward: GCA GAA GTT CCC TTC CCA TC<br>Reverse: GGA CAG GAA GAT GGT TCC AA
Ccr2	Forward: CCT TGG GAA TGA GTA ACT GTG TGA T<br>Reverse: ATG GAG AGA TAC CTT CGG AAC TTC
Nr4a1	Forward: AGC TTG GGT GTT GAT GTT CC<br>Reverse: AAT GCG ATT CTG CAG CTC TT
Vegfa	Forward: AAA AAC GAA AGC GCA AGA AA<br>Reverse: TTT CTC CGC TCT GAA CAA GG

Appendix table S5.

List of human real time PCR primers

Gene	Primer pair
RPS9	Forward: TGG TTT GCT TAG GCG CAG AC<br>Reverse: CCG CGG GGT CAC ATA AGT TT
VEGFA	Forward: CTA CCT CCA CCA TGC CAA GT<br>Reverse: GCA GTA GCT GCG CTG ATA GA
