# Supplementary material for: The chemokine receptor CX 3 CR1 coordinates monocyte recruitment and endothelial regeneration after arterial injury
Source: EMBO Mol Med. 2017 Dec 11;10(2):151–9. doi: 10.15252/emmm.201707502 (PMC5801509; doi:10.15252/emmm.201707502)
Supplement: Supplementary file 2 — Expanded View Figures PDF [file EMMM-10-151-s002.pdf]

Expanded View Figures

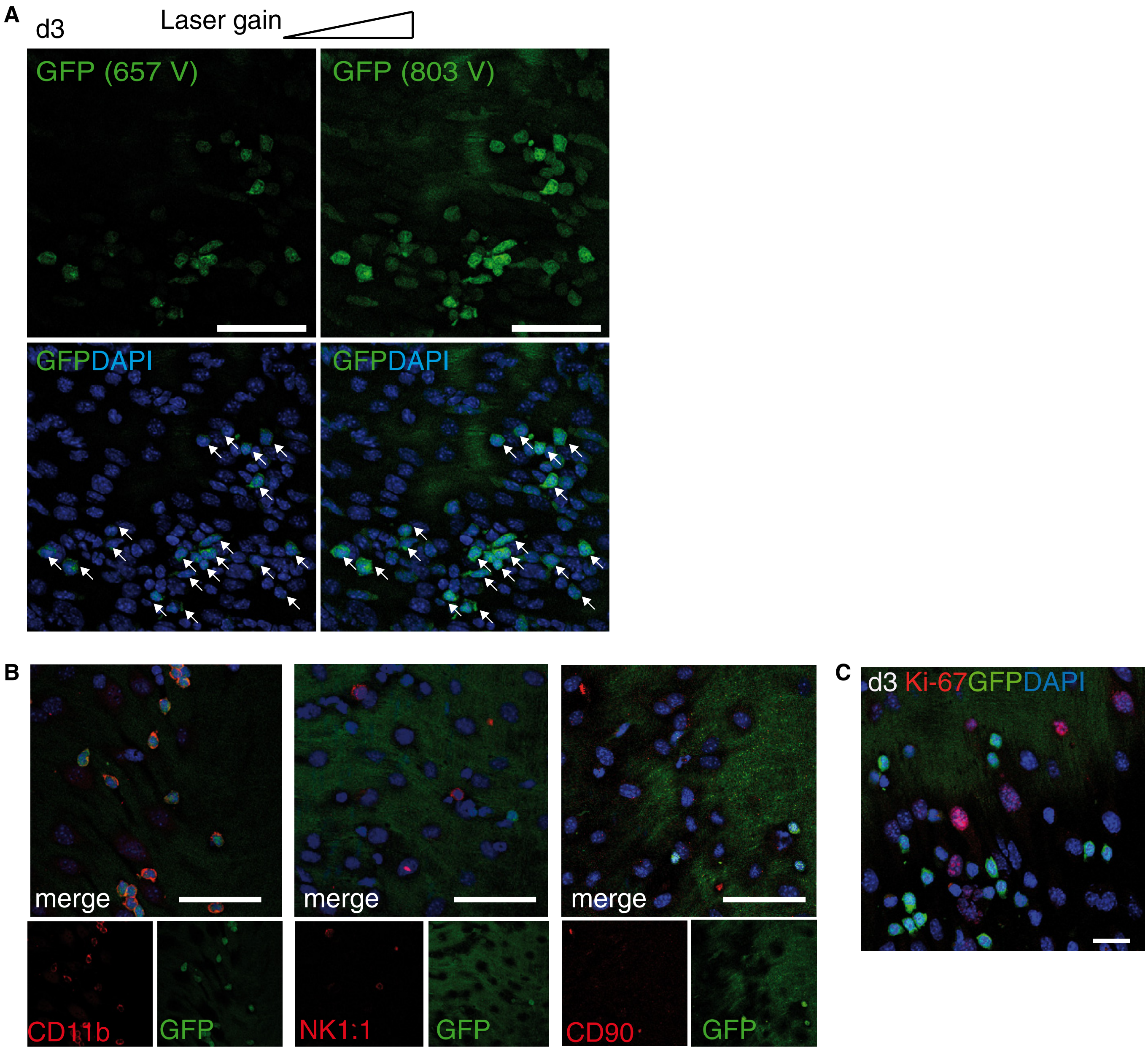

Figure EV1. Identification and characterization of GFP+ cells by confocal microscopy.

A Confocal images showing infiltration of wound area by Ly6C^{lo}GFP^{hi} cells (d2, static phase; d3, dynamic phase). Employing the technique of using high/low laser gain, we observed that the majority of the cells in the injured area are Ly6C^{lo}GFP^{hi} cells (white arrows) that fluoresce under low laser gain (657 V). Scale bars: 30 μm.

B Confocal images of carotid arteries 3 days postinjury stained for NK1.1 and CD90.2. The images in the lower inlay contain red (NK1.1/CD90) and green (GFP) channels. Scale bar: 75 μm.

C Confocal image of proliferation depicts Ki-67 expression in the proximal wound edge is specific to GFP^{-} cells (merge) (red, Ki-67; green, GFP; blue, DAPI). Scale bar: 150 μm.

 © 2017 The Authors

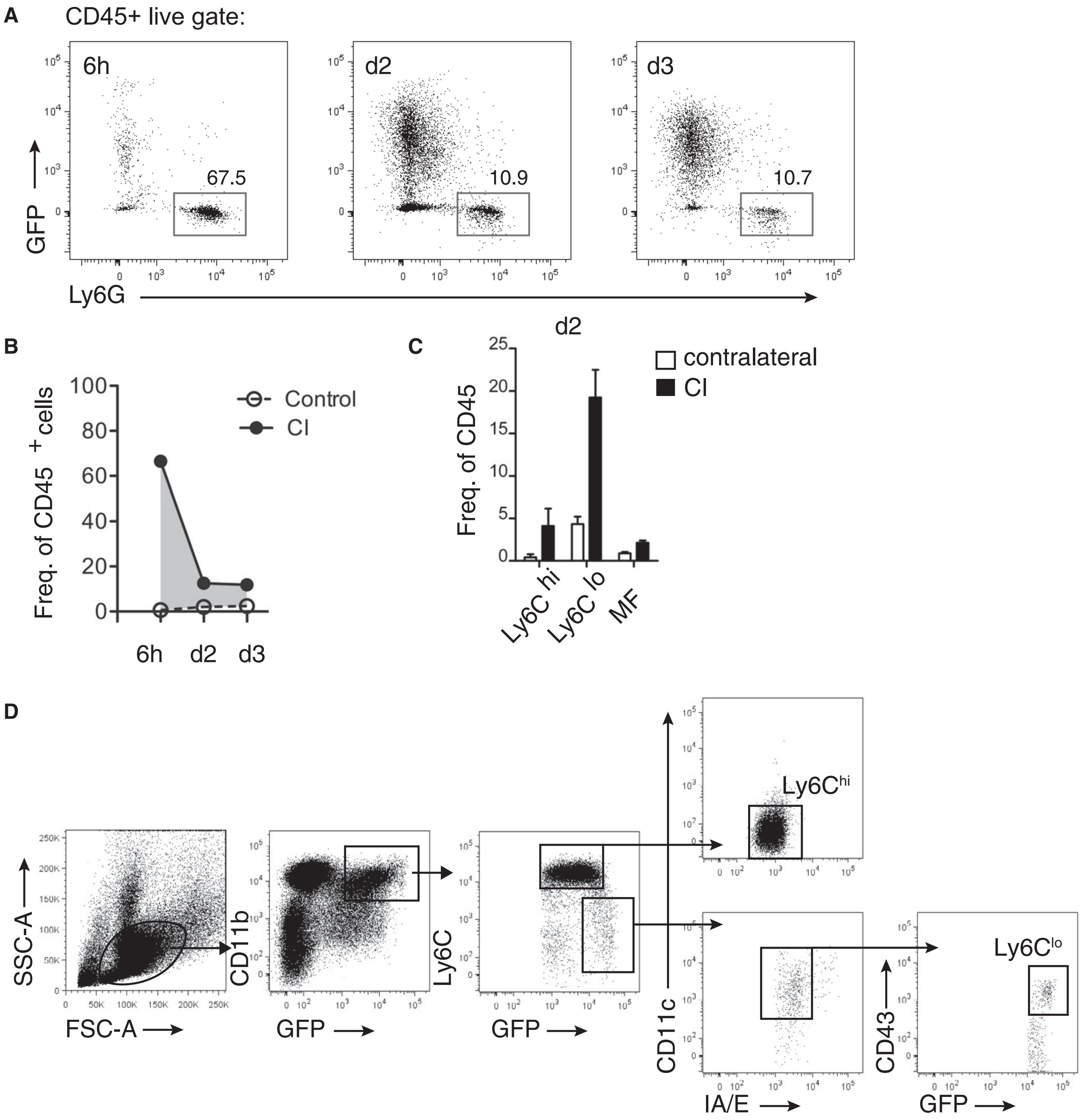

Figure EV2. Analysis of myeloid infiltration after carotid injury by flow cytometry.

A Representative flow cytometry plot depicting granulocyte ($CD11b^{+}GFP^{-}Ly6G^{+}$) infiltration 6 h, d2 and d3 after carotid injury.

B Quantitative analysis of myeloid cell infiltration depicted as frequency of $CD45^{+}PI^{-}$ (live) cells showing specific infiltration of $Ly6C^{hi}$, $Ly6C^{lo}$, and macrophages of injury site but not of contralateral uninjured carotid artery with $CD45^{+}$ cells. 6 h n = 3, d2 n = 5, d3 n = 3.

C Quantification of myeloid infiltration displayed as frequency of live cells showing that infiltration is specific to injured carotid at d2.

D Sorting strategy employed for isolation of $Ly6C^{hi}$ (blue) and $Ly6C^{lo}$ (black) murine monocyte subsets from bone marrow.

Data information: In (B, C), data are presented as mean ± SEM.

© 2017 The Authors

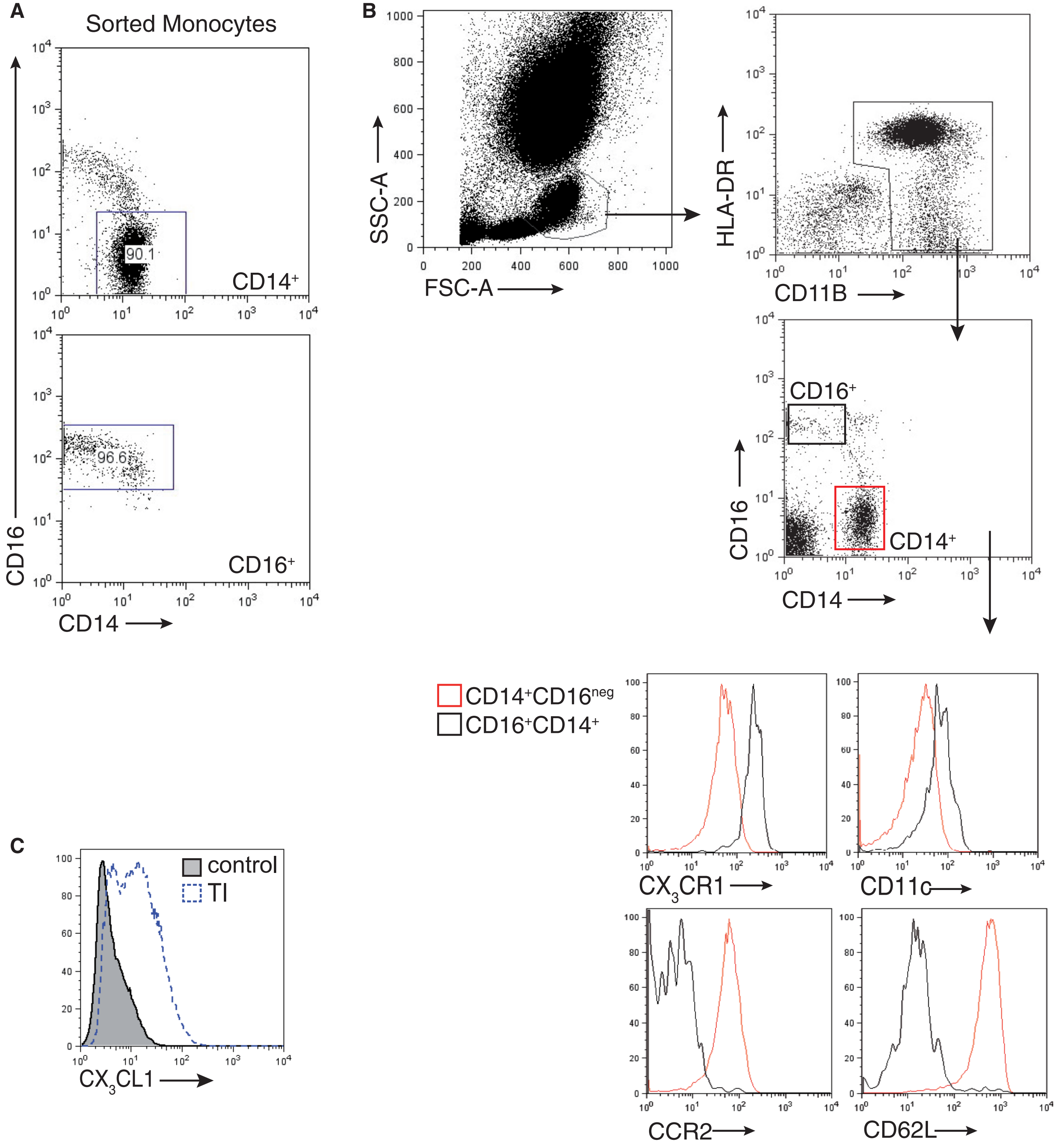

Figure EV3. Profile and isolation of human monocyte subsets and CX_3CL1 expression by EC.

- A Analysis of $CD14^{++}CD16^{neg}$ ($CD14^{+}$) and $CD14^{+}CD16^{++}$ ($CD16^{+}$) sorted monocytes after magnetic bead based sorting. Purity levels of > 90% for both sorted monocyte subsets.
- B Gating strategy for human monocyte subset characterization from PBMC (upper panel) and representative expression profiles from monocyte subsets (bottom) by flow cytometry.
- C Representative histogram depicting CX_3CL1 induction upon stimulation of HAECs with recombinant human TNF-α/IFN-γ (open dotted line). Representative histogram with unstimulated HAECs as a control (gray, shaded) is shown.

© 2017 The Authors
